# Related Mechanism of Limonene Improves LPS-Induced Neuroinflammation

**DOI:** 10.4014/jmb.2411.11053

**Published:** 2025-05-28

**Authors:** Yingjuan Jiang, Geng Liu, Qingqing Liu, Lanyue Zhang, Yanping Tan, Junlin Cen, Yan Zhao, Aiguo Li

**Affiliations:** 1 Department of General Medicine, Guangzhou Red Cross Hospital, Jinan University, Guangzhou 510220, P.R. China; 2 School of Biomedical and Pharmaceutical Sciences, Guangdong University of Technology, Guangzhou 510006, P.R. China; 3 Department of Neurology, Guangzhou Red Cross Hospital, Jinan University, Guangzhou 510220, P.R. China; 4 School of Public Health, Southwest Medical University, Sichuan 646000, P.R. China; 5 Guangzhou Institute of Traumatic Surgery, Department of Orthopedics, Guangzhou Red Cross Hospital, Jinan University, Guangzhou 510220, P.R. China

**Keywords:** Limonene, lipopolysaccharide, neuroinflammation, transcriptomics

## Abstract

Limonene, a component of volatile oils extracted from citrus plants, is known traditionally to treat inflammation. However, the anti-neuroinflammation efficacy and mechanism remain unclear. In this study, lipopolysaccharide was used as a neuroinflammatory inducer to investigate the mechanism of limonene in combating neuroinflammation in mice. Gas chromatography-mass spectrometry (GCMS) was used to identify the main components of *Citrus sinensis* (L.) Osbeck essential oil. A total of 21 compounds were identified in *Citrus sinensis* (L.) Osbeck essential oil, of which limonene, myrcene, and carene were the main components. Limonene was found to reduce LPS-induced neuroinflammatory responses in mice, as evidenced by decreased glial fibrillary acidic protein (GFAP) and ionized calcium-binding adapter molecule 1 (IBA-1) levels, along with suppressed expression of inflammatory cytokines. In addition, limonene improved the spatial memory and learning ability of mice caused by neuroinflammation, as well as neuronal death in the cerebral cortex and hippocampus. 3-(4,5-dimethylthiazol-2-yl)-2,5-diphenyltetrazolium bromide (MTT) results showed that limonene had no obvious toxicity to BV2 cells when the concentration was 4 mg/ml. Transcriptomic analysis revealed the effects of limonene on inflammatory response, immune regulation, and other signaling pathways. These results reveal that limonene may improve LPS-induced neuroinflammation by regulating microglia and astrocyte activation and inflammatory response and may be used as a drug for the treatment of neuroinflammation in the future.

## Introduction

Inflammation is a key feature of autoimmune disorders, with neuroinflammation primarily involving brain microglia. In pathological conditions, microglia are activated by external stimuli, producing inflammatory cytokines like prostaglandin E2, reactive oxygen species, and nitric oxide. Excessive cytokine release can damage or kill glial cells and neurons, reducing their numbers in the brain [1, 2]. Current research indicates a significant association between human neurodegenerative conditions and cerebral neuroinflammation, including Alzheimer's disease (AD), multiple sclerosis, and Parkinson's disease (PD) [3-5]. If untreated, protein aggregation (*e.g.*, β-amyloid and α-synuclein) activates glial cells, causing a feedback loop of inflammation that accelerates neuronal degeneration and behavioral changes [4, 6, 7]. These findings emphasize the importance of treating neuroinflammation early to prevent neurodegenerative diseases. Limonene has shown therapeutic effects in gastric and airway inflammation [8, 9], reducing the expression of interleukin-1 (IL-1), interleukin-6 (IL-6),(tumor necrosis factor-alpha) TNF-α, nuclear factor kappa-B (NF-κB), and Mpo, while upregulating the expression of Gpx [8]. However, research on its impact on neuroinflammation is limited, warranting further study. *Citrus sinensis* (L.) Osbeck essential oil, containing up to 45% limonene, is an ideal source for extraction [10, 11].

Lipopolysaccharide (LPS), a potent activator of the innate immune response, is known to elicit a robust inflammatory cascade in macrophage-like RAW 264.7 cells, characterized by the upregulation of nitric oxide, prostaglandin E2, and a suite of pro-inflammatory cytokines [12]. This inflammatory milieu is orchestrated by the heightened expression of key pro-inflammatory mediators, including TNF-α, IL-1β, and IL-6, which are central to the pathogenesis of neuroinflammatory disorders [12]. This process is crucial for studying the inflammatory response in the central nervous system (CNS) and for establishing models that mimic neuroinflammatory diseases.

In the context of neuroinflammation research, LPS plays a pivotal role in activating glial cells and inducing the release of cytokines, which are essential for understanding the pathogenesis of neuroinflammatory conditions. The relevance of LPS in glial cell activation and cytokine release is underscored by recent studies. For instance, study demonstrated that LPS stimulation of glial cells results in a robust upregulation of cytokines, which contribute to neuroinflammation and associated neurological disorders showing that LPS shifts glial cells to a more pro-inflammatory phenotype [13, 14]. This process results in the release of cytokines like TNF-α, IL-1β, and IL-6, which are key players in neuroinflammation [15]. Additionally, other studies showed that LPS can also affect the long-term behavior of glial cells, which has been demonstrated in studies exploring the long-term changes produced by microglia in response to LPS [16, 17]. These findings highlight the importance of LPS in modeling neuroinflammation and investigating the mechanisms underlying glial cell activation and cytokine-mediated neuroinflammatory processes.

TTP488, also known as Azeliragon, has been extensively studied for its ability to target the receptor for advanced glycation end products (RAGE), which plays a crucial role in the pathogenesis of various neurodegenerative diseases, including AD [18]. Recent studies demonstrated that TTP488 significantly reduced neuroinflammation in LPS-stimulated BV2 microglial cells by inhibiting the production of pro-inflammatory cytokines such as TNF-α and IL-1β [19, 20]. This finding aligns with our rationale for using TTP488 as a positive control in our study, as it provides a benchmark for the anti-inflammatory effects we aim to achieve with limonene.

Consequently, LPS serves as an effective model for stimulating glial cells to release excessive inflammatory cytokines, offering a platform to explore the array of proinflammatory and neurotoxic factors discharged by activated glial cells in mice [21-23]. To further substantiate our findings and provide a benchmark for the anti-inflammatory effects, we included TTP488 as a positive control in our study. In this investigation, mice subjected to the LPS model were utilized to assess the anti-inflammatory efficacy of limonene extracted from *C. sinensis* (L.) Osbeck. This was achieved through the analysis of mice behavior, neuronal cell count, gut microbiota, and the levels of ionized calcium-binding adaptor molecule-1 (IBA-1) and Glial fibrillary acidic protein (GFAP) in mice. These findings provide substantial theoretical and empirical support for the potential of limonene in mitigating and treating neural inflammation.

## Experimental Section

### Materials

The high-quality *C. sinensis* (L.) Osbeck was obtained from three distinct regions, meticulously cleansed, dried, mechanically pulverized, and sifted through a 0.45 mm sieve. Subsequently, the peel powder of *C. sinensis* (L.) Osbeck underwent a 3.5-h distillation process in 100°C water solvent at a material ratio of 1:15 to extract its volatile essential oil. The volatile essential oils were desiccated using anhydrous sodium sulfate and preserved in amber glass bottles at 4°C for subsequent experimental applications.

Preparation of drugs: *C. sinensis* (L.) Osbeck essential oil: 5 mg/ml (100 mg/kg, 10 g/0.2 ml gavage), Lim-L: 2.5 mg/ml (50 mg/kg, 10 g/0.2 ml gavage), Lim-H: 5 mg/ml (100 mg/kg, 10 g/0.2 ml gavage), LPS: 25 ug/ml (250 ug/kg, 10 g/0.1 ml injection), TTP488: 250 ug/ml (5 mg/kg, 10 g/0.2 ml gavage). All medications are dissolved in peanut oil.

### Gas Chromatography-Mass Spectrometer (GC-MS) Analysis

The essential oils extracted from *C. sinensis* (L.) Osbeck were subjected to GC-MS analysis using a GCMS-QP2010 PLUS (Shimadzu Co., Japan) with an FDT detector (EI mode, 70 EV). The analytical conditions were as follows: Helium was employed as the carrier gas at a flow rate of 0.87 ml/min; the column temperature was initially set to 90°C and gradually increase the temperature to 250°C at a rate of 5°C/min, held for 8 min; the ion source temperature was set to 200°C, and the transfer line was heated to 250°C. The identification of these compounds was established by comparing their retention times (RT), retention indices (RI), and mass spectra with those documented in the National Institute of Standards and Technology (NIST) Chemistry WebBook.

### Treatment of Laboratory Animals

**Fig. 1:**
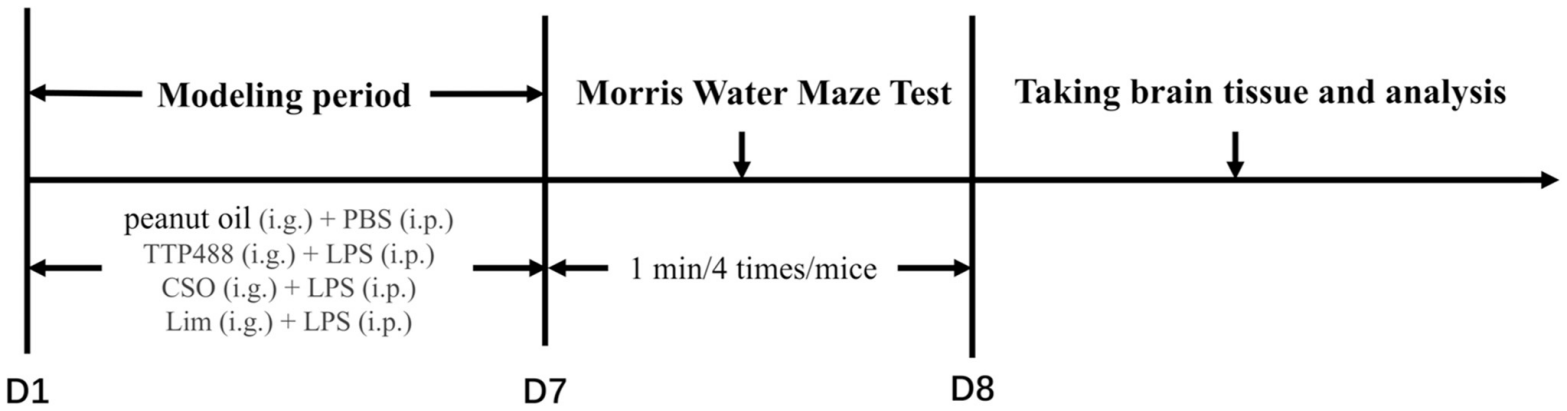
Schematic diagram of treatment and behavioral testing.

Adult male C57BL/6 mice (7–8 weeks, SPF grade) were purchased from the Guangdong Experimental Animal Center (China). All laboratory animals were kept under sterile conditions for seven days, with an environmental temperature of 23 ± 2°C, relative humidity of 75 ± 10%, and 12 h/12 h light/dark cycle (light on at 7:00, off at 19:00), and provided with standard laboratory water and food. The 48 male C57BL/6 mice were distributed into 6 cohorts, each comprising 8 mice: the control group, LPS group (250 μg/kg/d LPS), TTP488 group (250 μg/kg/d LPS + 5 mg/kg/d TTP488), CSO group (250 μg/kg/d LPS + *C. sinensis* (L.) Osbeck essential oil 100 mg/kg/d), Lim-L group (250 μg/kg/d LPS +50 mg/kg/d), and Lim-H group (250 μg/kg/d LPS + 100 mg/kg/d). Each group except the control group was injected with LPS, and the control group was given equal volume PBS. Following the modeling experiment, mice in the TTP488 group, the *C. sinensis* (L.) Osbeck essential oil group, and the limonene group underwent intragastric treatment for 7 days, while the remaining groups solely received peanut oil. The specific administration method and time are shown in Fig. 1.

### Morris Water Maze Test (MWM)

After 7 days of drug administration, the learning and memory abilities of mice in each group were evaluated by the MWM test. The water maze in this experiment was composed of a 120 cm-diameter plastic pool, filled with non-transparent water at 24 ± 1°C and a hidden platform with a diameter of 10 cm. Link it to the aerial camera of the Intelligent Video Tracking and Analysis System (TSE, Germany) and record all the experimental data in the pool. In the navigation test, the horizontal average center symmetry in the platform is divided into four quadrants, and the hidden platform is fixed at 1 cm underwater in the middle of a quadrant throughout the training cycle. The mice in each cohort underwent four daily training sessions, lasting one minute each, until they successfully located the concealed platform. The system mentioned above records escape latency in mice during these processes. Upon completion of the navigation test, the hidden platform was removed for the spatial probe test. The mice were placed in the swimming pool alone for 60 sec, and the percentage of time the mice spent in each quadrant and the frequency with which the mice passed through the platform area were calculated using aerial cameras.

### Nissl Staining

After the MWM test, all mice were euthanized, and their brains were dissected. From each group of 8 mice, 4 brain samples were soaked with 4% paraformaldehyde and fixed, 2 were preserved in liquid nitrogen at -80°C, and the remaining 2 were used for hippocampal homogenization. The brain tissue samples from both the control and experimental groups were washed with 0.01 M PBS and processed. The paraffin-embedded brain samples were sectioned using a rotating microtome to obtain continuous 5 μm thick coronal sections encompassing the hippocampal region. These sections were then stained with a 0.5% cresyl violet solution, washed off with distilled water, and dehydration using a series of graded ethanol solutions (70%, 95%, 100%). Tissue sections were clarified with xylene for 5 min and a glass coverslip was encapsulated with an encapsulant. Capture the image using a microscope. Normal neurons were quantified at 400x magnification. Three non-overlapping fields are randomly selected for each slice. Compare the number of Nistle bodies in the C2 and C3 regions of the hippocampus with neuronal apoptosis. Observe the apoptosis of neurons in the cortex and the number of Nistle bodies in normal neurons. Normal neurons have obvious Nistle granules and clear nuclei. Neuronal quantification in brain tissue was performed using Image-Pro Plus 6.0 software.

### Immunofluorescence Assays

The paraffin slices were baked in an oven at 62–65°C for 1 h, dewaxed, and hydrated in xylene-ethanol solution. Antigen retrieval was performed in citrate buffer (pH 6.0) in a microwave. After brief drying, a circle was drawn around the section using a tissue pen to prevent fluorescence quenching. The sections were blocked in 3% bovine serum albumin (BSA) at room temperature for 30 min and incubated with primary anti-IBA-1 antibody (1:100 in PBS) and primary anti-GFAP antibody (1:150 in PBS) at 4°C overnight and then with secondary anti-rabbit antibodies (1:100 in PBS) at room temperature for 50 min. To analyze, antifade DPI was mounted on the samples and an inverted fluorescence microscope (Carl Zeiss, Germany) was used to visualize the samples. Image Pro Plus 6.0 software was used to calculate the optical density of green and red fluorescence in the image.

### MTT Assay for Detecting Cell Viability

A single-cell suspension of mouse microglia (BV2) was prepared in the culture medium and incubated at 37°C in a 5% CO_2_ environment. Subsequently, 5,000 cells were seeded into each well of a 96-well plate, with a volume of 100 ul per well. After one day of cell culture, the original culture medium was replaced with culture medium containing 0.5, 1.0, 2.0, 4.0, and 8.0 mg/ml of *C. sinensis* (L.) Osbeck essential oil and limonene, respectively, and the cells were placed in an incubator and cultured for another day. On the third day, the original culture medium was discarded, and each well was washed three times with PBS. Then, 20 ul of 3-(4,5-dimethylthiazol-2-yl)-2,5-diphenyltetrazolium bromide (MTT) solution (5 mg/ml in PBS) was added to each well and incubated for 4 h. The culture was then terminated, and the supernatant carefully discarded. Subsequently, 150 ul of DMSO was added to each well and agitated for 10 min to ensure complete dissolution of the crystals. The absorbance of the samples at 490 nm wavelength was determined. The cell survival rate was calculated based on the measurement results.

### Detection of TNF-α and IL-1β by ELISA

The serum samples from each group of mice were collected following static coagulation. The protein concentration of the serum samples was assessed using the BCA protein concentration determination kit, following the provided instructions. Upon confirming the approximate concentration of the serum samples, the experiment proceeded in accordance with the guidelines of the Elisa detection kit to measure the concentration of TNF-α and IL-1β in the blood samples.

### RNA-Seq

Total RNA (500 ng per sample) was extracted and used to construct sequencing libraries with the TruSeq RNA library preparation kit (Illumina, USA), following the manufacturer's instructions. Sequencing was performed on the HiSeq 2000 (Illumina) platform in a paired-end mode with a read length of 2 × 100 bp. Transcriptome sequencing data were obtained through high-throughput sequencing of the raw sequences. Outlier samples in each group were detected by principal component analysis (PCA), and sample clusters with high similarity were identified, after which the read counts for genes were determined. DESeq2, DEGseq, and edgeR were used to identify differentially expressed genes between groups. Venn analysis was performed to determine the distribution of genes in each gene set. Finally, functional enrichment analysis was conducted for genes in the gene sets based on Gene Ontology (GO) and Kyoto Encyclopedia of Genes and Genomes (KEGG) annotations. Fisher's exact test was used for data evaluation.

### Statistical Analysis

The data were expressed as the mean ± standard deviation (SD) and analyzed by one-way ANOVA with Duncan's similarity test using Graph Pad Prism (V8.0.2.263). Statistical significance was set at *P* < 0.05.

## Result

### Analysis of *Citrus sinensis* (L.) Osbeck Essential Oil

The essential oil was extracted from *C. sinensis* (L.) Osbeck to obtain the essential oil at the interface temperature of 280°C. The main components of *C. sinensis* (L.) Osbeck essential oil were identified by GCMS as 21 species, accounting for 99.94%, primarily including limonene (81.30%), myrcene (6.83%), and carene (2.81%). The total monoterpenoids accounted for 98.14%. Oxygenated monoterpene was 3.12% and total sesquiterpenoids was 1.34%. The primary components and their respective percentages in *C. sinensis* (L.) Osbeck are presented in Table 1.

### The Behavioral Effects of *Citrus sinensis* (L.) Osbeck Essential Oil on LPS-Induced Neuroinflammation Model Mice

**Fig. 2:**
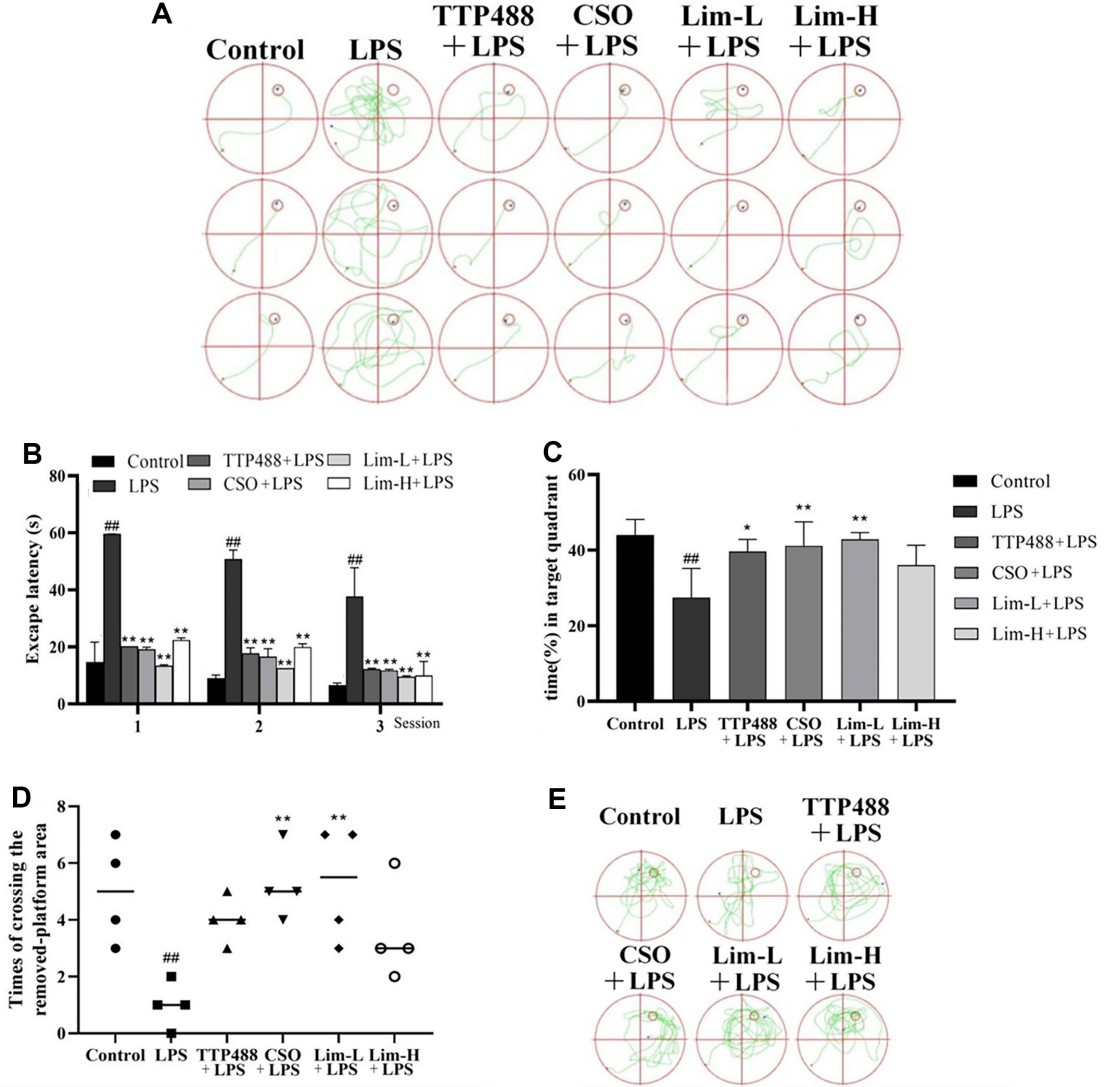
Illustrates the impact of *Citrus sinensis* (L.) Osbeck essential oil on LPS-induced memory damage and associated brain nerves. (**A**) Directional navigation (hidden platform) tests in mice on paths. (**B**) Escape latency in mice during directional navigation (hidden platform) experiments. (**C**) In the space exploration experiment, the percentage of time the mice spent searching in the quadrant where the target was located. (**D**) The times the mice crossed the removed platform. (**E**) The motion trajectory of mice in the space exploration test. Data was represented using mean ± SD (*n* = 8 mice per group). In comparison to the control group, significance denoted as #*p* < 0.05, ##*p* < 0.01. In comparison to the LPS group, significance denoted as *p < 0.05, **p < 0.01. CSO: *Citrus sinensis* (L.) Osbeck essential oil group.

Based on current research, it has been observed that systemic administration of LPS in mice may result in diminished cognitive abilities related to learning and memory [24, 25]. In this study, the Morris water maze (MWM) was utilized to assess the impact of limonene on the learning and memory capabilities of mice in an LPS injection model, with control, positive, and *C. sinensis* (L.) Osbeck essential oil groups established to enhance the reliability and precision of the experiment. During the spatial navigation experiment (hidden platform) (Fig. 2B), it was noted that the escape latency was longer in the LPS group compared to the control group, and the difference was statistically significant (*p* < 0.01), indicating the successful establishment of the LPS injection model in mice.In contrast to the LPS group, limonene notably diminished the escape latency of mice across the first, second, and third days at various time intervals, with an effect akin to TTP488 treatment, demonstrating significant disparities (*p* < 0.01). The lower concentration of limonene exhibited greater efficacy in counteracting LPS induction compared to the higher concentration. The therapeutic impact of the CSO group fell between the high and low concentrations, or lower, but were significantly different from those in the LPS group (*p* < 0.01). The trajectory of the mice in the experiment aligned with the escape latency time (Fig. 2A).

Report on Space Exploration Experiment: The mice in the LPS group exhibited reduced time spent in the target quadrant and fewer instances of passing through the removed platform compared to the control group mice (Fig. 2C and 2D), with significant disparities (*p* < 0.01), affirming the successful establishment of the LPS injection model in the mice. Compared with LPS group, the mice treated with a low concentration of limonene showed a notably increased proportion of time spent in the target quadrant, and the intrinsic difference was significant (*p* < 0.01). The therapeutic impact of limonene surpassed that of TTP488. The trajectory of the mice in the experimental report corroborated these findings (Fig. 2E).

### Cell Viability Rate of *Citrus sinensis* (L.) Osbeck Essential Oil and Limonene

**Fig. 3:**
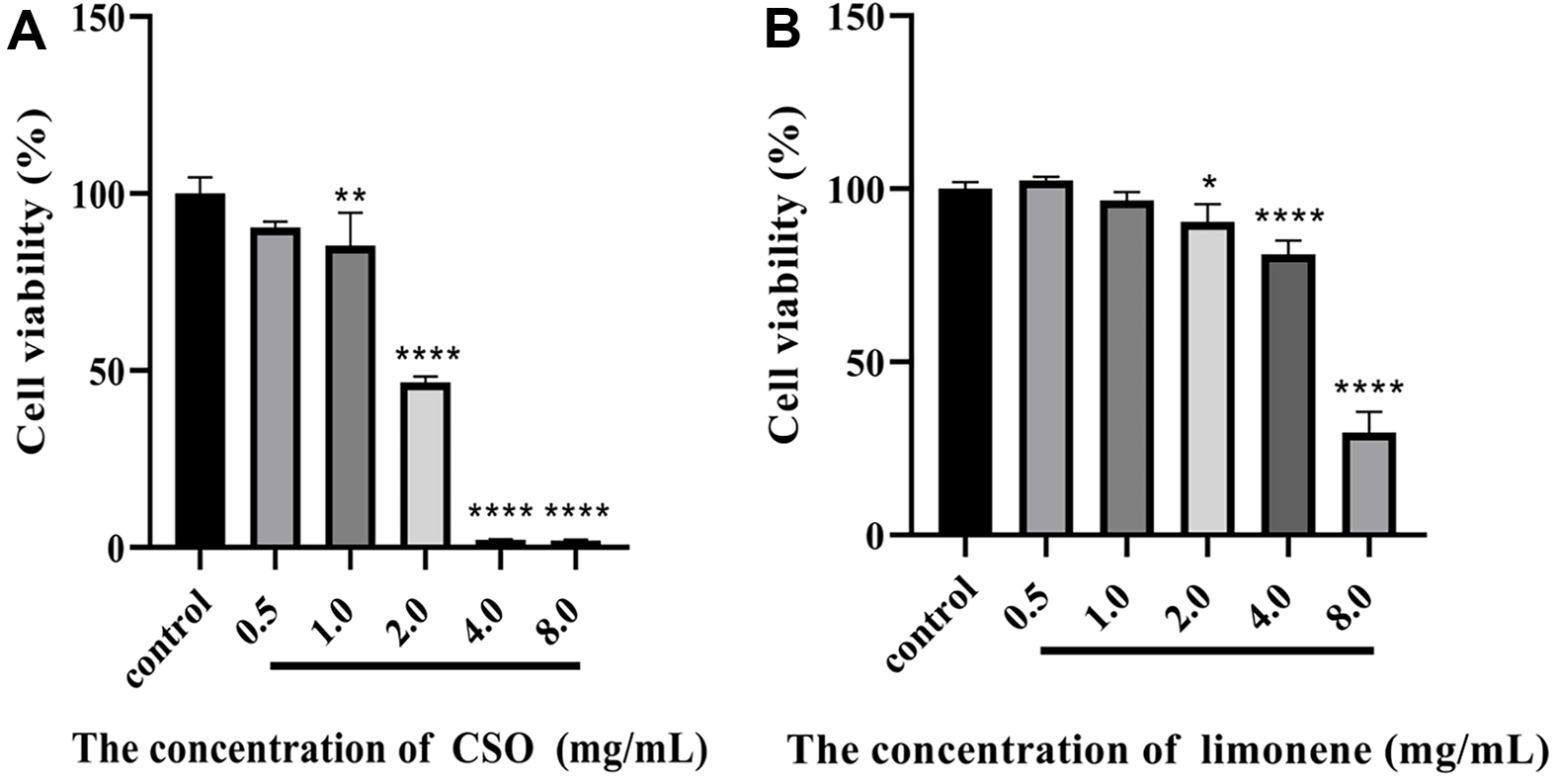
Effects of *Citrus sinensis* (L.) Osbeck essential oil and limonene in the BV2 cell viability. (**A**) MTT assay was used to evaluate the cell viability of *Citrus sinensis* (L.) Osbeck essential oil at different concentrations. (**B**) MTT assay was used to evaluate the cell viability of limonene at different concentrations. Data was represented using mean ± SD (*n* = 6 per group). In comparison to the LPS group, significance denoted as *p < 0.05, **p < 0.01 and ****p < 0.0001. CSO: *Citrus sinensis* (L.) Osbeck essential oil group.

Drug cytotoxicity detection can reveal whether a drug has a toxic effect on cells and the degree of such toxic effect, so as to help eliminate interfering factors and accurately analyze the effects of drugs in LPS models. As shown in Fig. 3A, *C. sinensis* (L.) Osbeck essential oil showed a significant toxic effect on BV2 cells at a concentration of 2.0 mg/ml (*p* < 0.0001), and limonene showed a significant toxic effect on BV2 cells at a concentration of 8.0 mg/ml (*p* < 0.01) in Fig. 3B. The results confirmed that limonene, as the main component of *C. sinensis* (L.) Osbeck essential oil, was safe for the proliferation of BV2 cells at a concentration below 8.0 mg/ml.

### Therapeutic Effects of Limonene on LPS-Induced Neuronal Injury

**Fig. 4:**
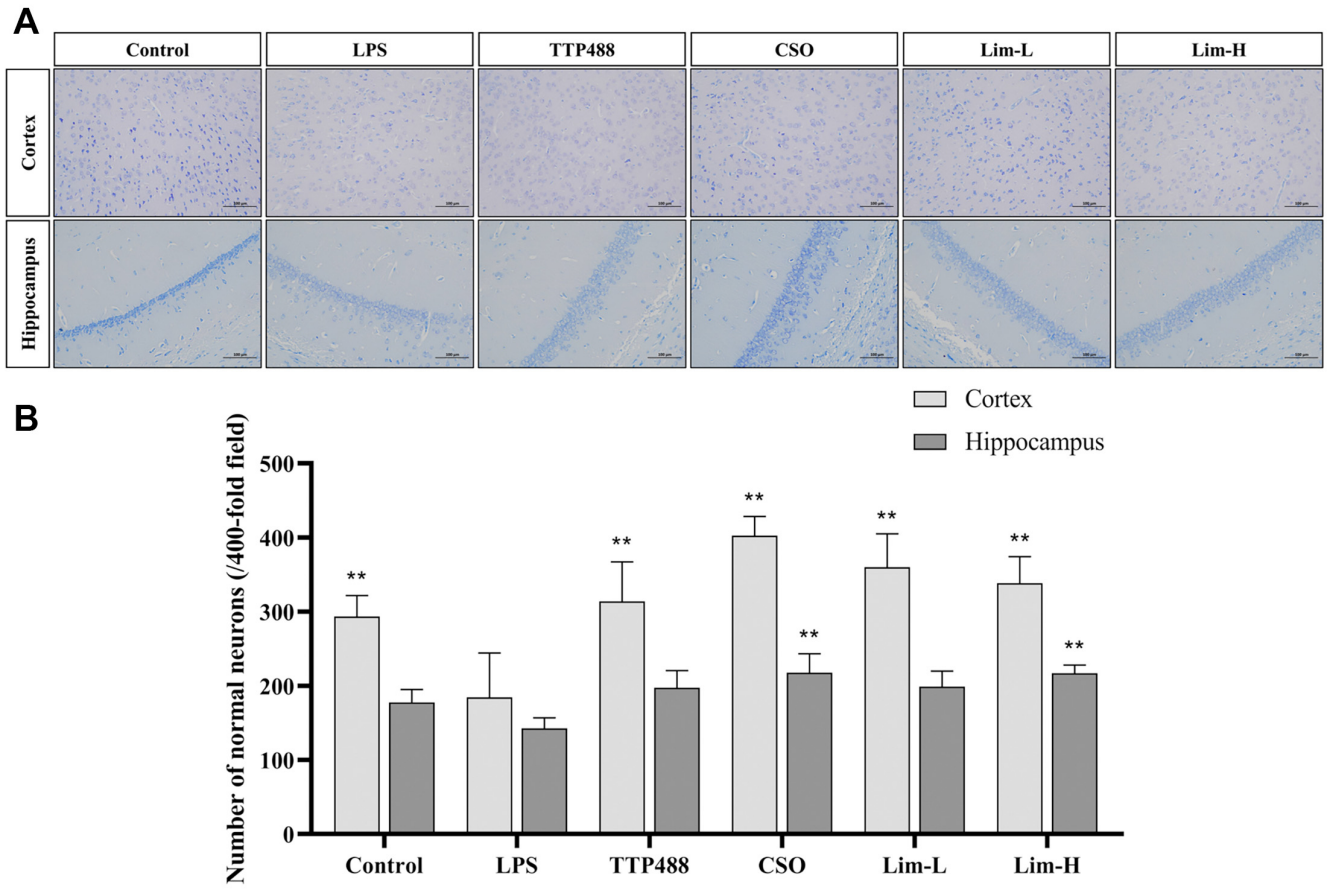
Illustrates the impact of limonene on LPS-induced neuronal injury in mice. (**A**) Visualization of representative images of Nissl staining in the cortex and hippocampus of each group of mice. (**B**) The number of normal neurons in the cortex and hippocampus of mice in each group was quantified, with each bar representing 100 μm. Data was represented using mean ± SD (*n* = 8 mice per group). In comparison to the LPS group, significance denoted as **p < 0.01. CSO: *Citrus sinensis* (L.) Osbeck essential oil group.

To further validate the neuroprotective effects of limonene on mice, Nissl staining was employed to assess the therapeutic impact of limonene on LPS-induced histopathological alterations in neurons. The results of the experiment revealed a notable decrease in the number of neurons in the cerebral cortex of LPS-treated mice compared to the control group, with a particularly pronounced effect and significant disparities (*p* < 0.01). In the cerebral cortex, each treatment group exhibited a marked increase in the number of normal neurons, with significant differences (*p* < 0.01). The therapeutic efficacy of limonene and the CSO group surpassed that of the TTP488 group, with the CSO group demonstrating the most potent therapeutic effect. As shown in Fig. 4, in the hippocampus, the treatment groups displayed varying abilities to augment the number of normal neurons, with significant differences between the CSO group and the Lim-H group (*p* < 0.01).

### Immunofluorescence Analysis of the Effect of Limonene on LPS Model Mice

**Fig. 5:**
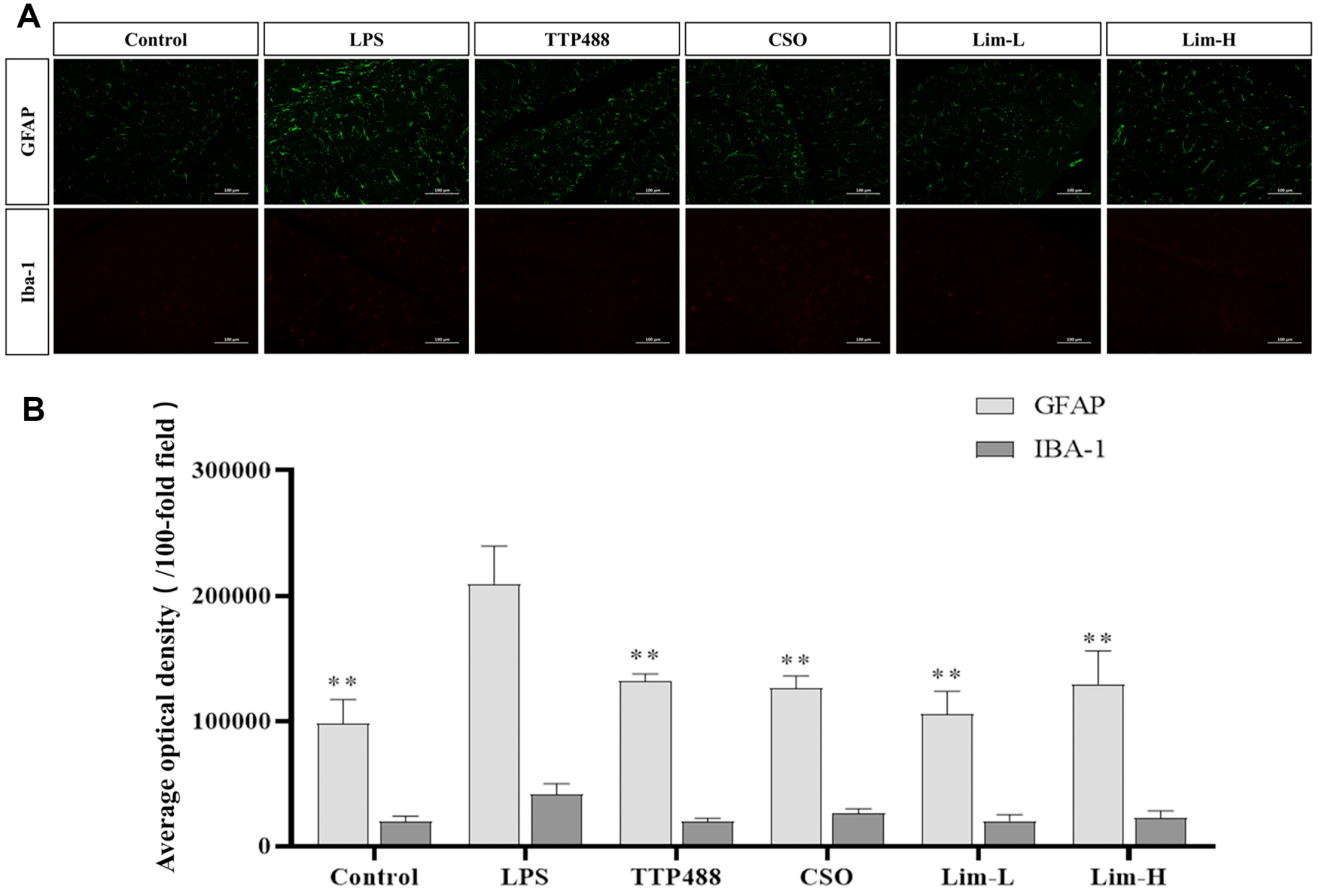
Impact of Limonene on LPS-Induced Neuroinflammation in Cortex and Hippocampus of C57 Mice. (**A**) Immunofluorescence assessment of GFAP and IBA-1 content, evaluating astrocyte (GFAP positive cells) and microglia (Iba-1 positive cells) in each group, scale bar = 100 μm. (**B**) Immunofluorescence measurement of the proportion of GFAP and IBA-1. Data was represented using mean ± SD (*n* = 8 mice per group). In comparison to the LPS group, significance denoted as **p < 0.01. CSO: *Citrus sinensis* (L.) Osbeck essential oil group.

Neuroinflammation is an immune response activated by microglia and astrocytes in the CNS. Astrocytes, through the upregulation of GFAP, contribute to the formation of the glial scar and modulate inflammation [26]. Microglia are rapidly activated in response to LPS stimulation, leading to the upregulation of IBA-1, which serves as a marker for microglial activation but also participates in the cellular mechanisms underlying the neuroinflammatory response [27]. The immunofluorescence assessment of these cells provides a visual and quantitative measure of their activation state, which is indicative of the neuroinflammatory environment. In mice models of LPS-induced neuroinflammation, overactivation of microglia and astrocytes is known to promote significantly increased levels of GFAP and IBA-1. Hence, the objective of this investigation was to explore the suppressive impact of limonene on LPS-triggered microglial and astrocytes activation in mice through the quantitative assessment of GFAP and IBA-1 levels. By observing Fig. 5A and Fig. 5B, it is found that compared with the control group, the levels of GFAP and IBA-1 in LPS group were significantly increased, and the variation of GFAP was significantly different (*p* < 0.01), indicating the successful induction of neuroinflammation in the mice. Comparison with the control group, the GFAP assay demonstrated a substantial reduction in GFAP content in the treatment group, (*p* < 0.01). The most effective treatment was observed in the Lim-L group. The IBA-1 assay indicated a reduction in IBA-1 content in the treatment group, with the most effective treatment observed in the TTP488 group. Notably, in the limonene treatment group, the lower concentration yielded superior results compared to the higher concentration. Khan *et al*.'s study confirmed that glial cell activation was mainly mediated by TLR4 [28]. As a receptor for LPS, TLR4 was a key and common mediator of innate inflammation. TLR4 produced an immune response by recognizing pathogens, which was usually manifested as increased expression of GFAP (astrocyte marker) in astrocytes and IBA-1 (microglia marker) in microglia. TLR4 triggered a series of cellular responses by activating the downstream NF-κB signaling pathway, mainly including the release of inflammatory factors (such as cytokines, chemokines, and prostaglandins).

### Inflammatory Cytokines Concentration in Blood

**Fig. 6:**
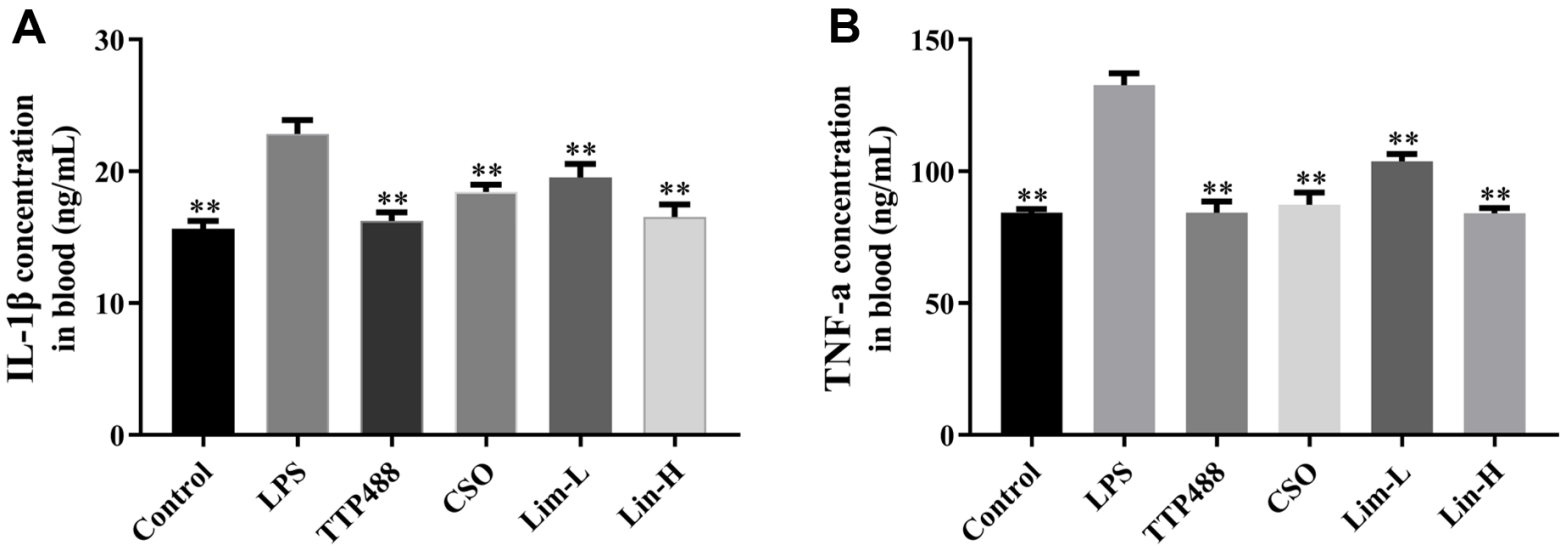
ELISA analysis was performed to ascertain the levels of TNF-α and IL-1β in the blood samples from the specified groups of mice. The data presented reflects the mean ± SD (*n* = 8 mice per group). In comparison to the LPS group, significance denoted as **p < 0.01. CSO: *Citrus sinensis* (L.) Osbeck essential oil group.

To further confirm that limonene can improve neuroinflammation in mice, this experiment used ELISA to determine the inflammatory factor of TNF-α and IL-1 β levels in the blood of mice. The experimental data shows that, under significant differences (*p* < 0.01), compared with the Control group, the LPS model group was successfully modeled. As shown in Fig. 6A and Fig. 6B, the TTP488 group, CSO group, Lim-L, and Lim-H groups showed varying degrees of decrease in the concentration of two inflammatory factors compared with LPS model group. It can be clearly seen that the TTP488 group showed the most significant decrease, followed by the COS group.

### RNA-Seq Analysis of Limonene

**Fig. 7:**
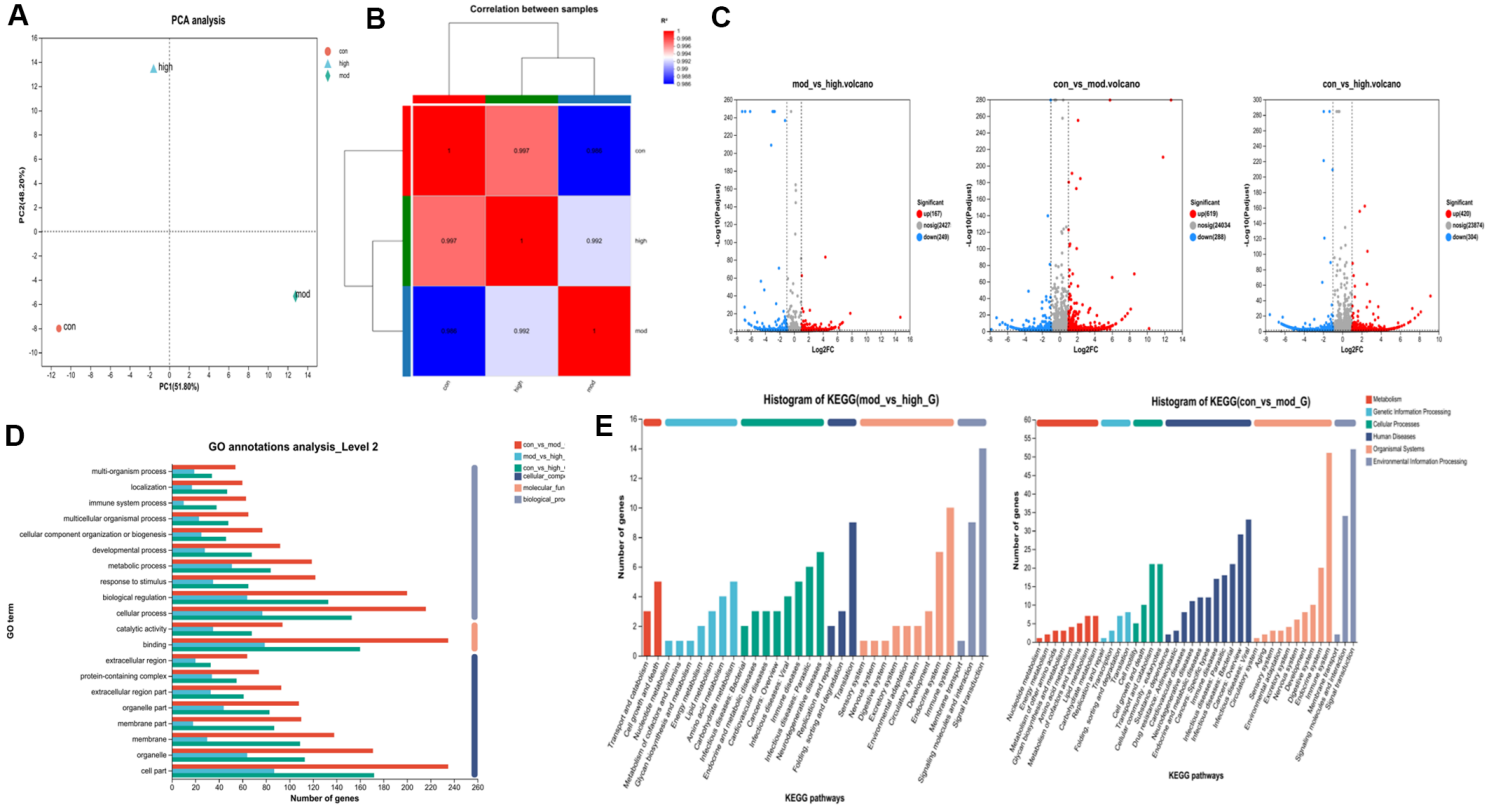
(A) PCA diagram; (B) Sample correlation. Higher absolute value of R2 indicating a stronger correlation between the paired samples. (**C**) Volcanic plot of mod-vs-high, con- vs -mod, and con- vs-high (*p* < 0.05). (**D**) GO functions of the con-vs-mod-G group, mod-vs-high-G group and con-vs-high-G group. (**E**) KEGG enrichments of the mod-vs-high-G group and con- vs -mod-G group.

Principal component analysis (PCA) was employed to assess the overall genetic variance among samples and the level of resemblance within sample groups. In Fig. 7 (A and B), there was a large difference between the samples in each group, indicating that there were significant differentially expressed genes after modeling, which could be analyzed in the next step. The default genetic screening threshold is set at *p* < 0.05, | log 2 FC |>1.2. The volcano plot in Fig. 7C illustrated the overall gene count detected in the differential group, along with the number of significantly upregulated and downregulated differential genes. It indicated that the gene difference multiple in con- *vs* -mod exhibits the highest level of upregulation, while the gene significance in con- vs-high is more pronounced, and the majority of genes in mod-*vs*-high are notably significant.

Gene Ontology (GO) annotation was conducted for all genes and differentially expressed genes, and the annotation results were shown in Fig. 7D. In the biological process category, the majority of genes were annotated on GO terms related to binding. In the cell component category, GO terms related to cell part were annotated with the most genes. Among the molecular functional categories, GO terms associated with cellular process exhibited the highest number of annotated genes. The Kyoto Encyclopedia of Genes and Genomes (KEGG) analysis depicted in Fig. 7E reveals a wealth of enriched signaling pathways in each control group, predominantly linked to processes such as inflammatory responses, immune modulation, hormone synthesis and metabolism, and vitamin regulation. The graphical representations indicate the aggregation of numerous genes involved in signaling molecules and their effects, neurodegenerative diseases, and the immune system.

## Discussion

The reduction in TNF-α, IL-1β, GFAP, and IBA-1 indicates suppressed inflammation, which is crucial for initiating neural recovery [29, 30]. Initially triggered by peripheral immune challenges like LPS, these factors can cause neuronal and synaptic damage. In our study, limonene mitigated the harmful effects of chronic inflammation on neuronal integrity and synaptic function by downregulating these markers. The improvements in spatial memory and learning, as demonstrated by the Morris Water Maze test, reflect the resolution of neuroinflammation and subsequent repair of neuronal and synaptic damage. This behavioral recovery is underpinned by limonene's neuroprotective effects, which alleviate inflammation and support cognitive function. Essentially, the decrease in pro-inflammatory mediators and glial cell activation markers signifies a shift towards a more resolving phenotype, facilitating the restoration of neuronal and synaptic health, and ultimately improving cognitive and behavioral deficits in mice. From micro-scale inflammation to macro-scale behavioral recovery, limonene exhibits complex interactions. And compared with other anti-inflammatory compounds, there was a similar mechanism of action. For example, curcumin inhibited migration, autophagy, and pro-inflammatory mediators through the AMPK signaling pathway in LPS-activated astrocytes, reduced PI3K/Akt phosphorylation and NF-κB activation in LPS-activated microglia, and had a preventive effect on LPS-induced acute neuroinflammation and long-term memory impairment. Another anti-inflammatory compound, resveratrol, effectively improved LPS-induced spatial and working memory impairment, reduced hippocampal glial density and neuronal loss in LPS-injected mice, and inhibited LPS-induced upregulation of NF-κB, IL-6, IL-1β, and GFAP in the hippocampus of mice [31-34].

In this study, low concentration limonene (Lim-L: 50 mg/kg) showed better therapeutic effects than high concentration limonene (Lim-H: 100 mg/kg). The pharmacokinetic properties of limonene in vivo may also influence its dose-response relationship. At lower doses, limonene may bind more effectively to specific receptors or enzymes to exert its pharmacological effects, while higher doses may exceed the saturation point of the receptor, resulting in effects that do not increase with dose. Low concentrations of limonene may be more in line with its natural metabolic rate in the body, thus maintaining effective concentration and activity in the organism, while high concentrations of limonene may lead to increased metabolic burden. This observation needs to be further explored and the complex biological effects of limonene, especially the underlying mechanisms regarding drug administration strategies, need to be considered in the study and application of limonene in order to better understand and utilize the pharmacological effects of limonene [35]. The GO annotation revealed that most genes may be involved in key processes such as cell signaling and molecular recognition. The decline in GFAP after limonene treatment suggests that limonene may reduce astrocyte activation, a key event in neuroinflammation. Secondly, decreases in TNF-α and IL-1β further support the anti-inflammatory effects of limonene. These cytokines play a central role in the inflammatory response, and their downregulation suggests that limonene may inhibit the production of inflammatory mediators. KEGG analysis revealed the effects of limonene on inflammatory response, immune regulation and other signaling pathways. The enrichment of these pathways suggests that limonene may exert its anti-inflammatory effects by modulating these key biological processes and influencing the NF-κB signaling pathway [36, 37].

Limonene also exhibited significant anti-inflammatory effects in various inflammation models. In LPS-induced Parkinson's disease, limonene significantly reduced oxidative nitrative stress, malondialdehyde (MDA) and nitrite levels, and increased catalase, glutathione (GSH), and superoxide dismutation enzyme (SOD) levels [38]. Additionally, limonene reduced the production of pro-inflammatory cytokines by inhibiting NF-κB and MAPK activation, thereby alleviating the inflammatory response of lipopolysaccharide-induced acute lung injury [39]. Furthermore, limonene improved depression in maternally separated mice and reduced neuroinflammation by lowering hippocampal nitrite levels and the expression of IL-1β and TNF-α [40]. These results suggested that limonene had potential neuroprotective effects through mechanisms such as antioxidant activity, anti-inflammatory effects, and modulation of immune responses. This study confirmed that limonene improved the learning ability and spatial memory impairment of LPS-induced neuroinflammatory mice, reduced the levels of IBA-1 and GFAP to reduce glial activation, and downregulated the levels of pro-inflammatory factors (TNF-α and IL-1β) to inhibit the inflammatory response. Transcriptome analysis confirmed that limonene exerted anti-neuroinflammatory effects by regulating signaling pathways such as endocrine, immune system and signal transduction, which has not been reported in previous studies. However, although this study explored the role of limonene in reducing inflammation and oxidative stress, it did not involve a wider range of signaling pathways, especially the TLR4 (Toll-like receptor 4) signaling pathway. Collectively, these results underscore the promise of limonene as a natural therapeutic agent for mitigating neuroinflammation and suggest that lower concentrations may be more efficacious, possibly due to optimized pharmacokinetic properties and reduced metabolic burden. Notably, lower concentrations of limonene proved to be more efficacious than higher concentrations, indicating that minimal quantities of active limonene molecules are sufficient to yield favorable therapeutic effects.

## Conclusion

In conclusion, the present study provides evidence that limonene, a major component of citrus essential oil, exerts significant therapeutic effects against LPS-induced neuroinflammation. Our findings demonstrate that limonene, particularly at a low concentration of 50 mg/kg (Lim-L), effectively reverses neuroinflammation in mice by reducing the levels of pro-inflammatory cytokines TNF-α and IL-1β and downregulating the activation markers IBA-1 and GFAP in glial cells. The restoration of neuronal function, as indicated by the increase in Nissl bodies, further supports the neuroprotective potential of limonene. After limonene treatment, the results of the Morris water maze test showed improved spatial memory ability and behavioral performance of the mice. The transcriptomic analysis enriches our understanding by highlighting the modulation of "endocrine, immune system" and "signal transduction" pathways, which are pivotal in neuroinflammatory processes.

## Figures and Tables

**Table 1:** Retention indices (RI) and relative content (%) of each compound identified in the essential oils of *Citrus sinensis* (L.) Osbeck.

No	Compounds ^i^	RI ^ii^	Exp. RI	Ref.	Relative content
					*Sichuan Turmeric*
1	α-Pinene	985.03	933	A	1.71%
2	Sabinene	1015.29	977	B	0.25%
3	β-pinene	1016.08	972	A	0.60%
4	Isooctane	1024.20			0.01%
5	Myrcene	1025.80	985	A	6.84%
6	α-phellandrene	1036.31	1003	B	0.23%
7	Octanal	1037.90	1004	B	0.35%
8	carene	1040.29	1011	C	2.81%
9	α-Terpinene	1044.59	1018	C	0.47%
10	limonene	1062.42	1031	C	81.30%
11	γ-Terpinene	1087.26	1062	C	0.49%
12	2-Carene	1113.99	1001	C	0.14%
13	Terpinolene	1117.66	1088	C	0.25%
14	Linalool	1139.67	1098	C	0.73%
15	[Bicyclopentyl]-2-one	1144.84			0.25%
16	4-terpineol	1190.63	1177	C	0.95%
17	dihydrocarveol	1210.79			1.11%
18	β-Caryophyllene	1349.24	1418	C	0.11%
19	Germacrene D	1389.49	1480	C	0.02%
20	valencene	1395.44	1479	D	1.22%
21	isopulegol	1532.08	1156	E	0.08%
Total identified/%					99.94%
Total monoterpenoids/%					98.14
Oxygenated monoterpenes/%					3.12
Total sesquiterpenoids/%					1.34
Others/%					0.01

^I^The compounds eluted from methyl silicone capillary columns (30 m × 0.25 mm, 0.25-μm film thickness) are arranged sequentially.

^ii^Retention index (RIs) on the same capillary column with methylsilicone as stationary phase (relative to normal alkanes (C_6_-C_40_)).

^A^Allegrone, Belliardo, *et al*., 2006; ^B^Asuming, Beauchamp, *et al*., 2005; ^C^Adams, González Elizondo, *et al*., 2006; ^D^Angioni, Barra, *et al*., 2006; ^E^Jalali-Heravi, Zekavat, *et al*., 2006.
